# The Effect of a Pro-Breastfeeding and Healthy Complementary Feeding Intervention Targeting Adolescent Mothers and Grandmothers on Growth and Prevalence of Overweight of Preschool Children

**DOI:** 10.1371/journal.pone.0131884

**Published:** 2015-07-10

**Authors:** Renata Schwartz, Álvaro Vigo, Luciana Dias de Oliveira, Elsa Regina Justo Giugliani

**Affiliations:** 1 Graduate Program in Child and Adolescent Health, Universidade Federal do Rio Grande do Sul, Porto Alegre, Rio Grande do Sul, Brazil; 2 Department of Statistics, Universidade Federal do Rio Grande do Sul, Porto Alegre, Rio Grande do Sul, Brazil; 3 Department of Nutrition, Universidade Federal do Rio Grande do Sul, Center for Food and Nutrition Studies (CESAN), Hospital de Clínicas de Porto Alegre, HCPA, Porto Alegre, Rio Grande do Sul, Brazil; University of Ottawa, CANADA

## Abstract

**Introduction:**

The pattern and duration of breastfeeding (BF) and the age at onset of complementary feeding, as well as its quality, have been associated with the prevalence of overweight in childhood.

**Objective:**

To assess the effect of a pro-BF and healthy complementary feeding intervention, targeted to adolescent mothers and maternal grandmothers, on growth and prevalence of overweight and obesity in children at preschool age. This intervention had a positive impact on duration of BF and timing of onset of complementary feeding.

**Methods:**

This randomized clinical trial involved 323 adolescent mothers, their infants, and the infants’ maternal grandmothers, when they cohabited. Mothers and grandmothers in the intervention group received counseling sessions on BF and healthy complementary feeding at the maternity ward and at home (7, 15, 30, 60, and 120 days after delivery). When children were aged 4 to 7 years, they underwent anthropometric assessment and collection of data on dietary habits. Multivariable Poisson regression with robust estimation was used for analysis.

**Results:**

BMI-for-age and height-for-age were similar in the intervention and control groups, as was the prevalence of overweight (39% vs. 31% respectively; p=0.318). There were no significant between-group differences in dietary habits.

**Conclusion:**

Although the intervention prolonged the duration of exclusive BF and delayed the onset of complementary feeding, it had no impact on growth or prevalence of overweight at age 4 to 7 years.

**Trial Registration:**

ClinicalTrials.gov NCT00910377

## Introduction

Human dietary habits are undergoing significant changes, with increased intake of industrialized products, a reduction in fresh fruit and vegetable consumption, and more frequent meals outside the home. These changes have led to an inversion in the nutritional pattern of the population, i.e., the nutrition transition. Currently, the increasing prevalence of overweight in many parts of the world, including in children and adolescents, is a big concern [1–3]. According to the World Health Organization (WHO), the prevalence of obesity in under-fives had increased from 4.2% in 1990 to 6.7% in 2010. If the trend continues, this prevalence is expected to rise to 9.1% by 2020 –a relative increase of 36% from 2010 levels [4].

In Brazil, the prevalence of obesity in under-fives is 7.3% [5]. In the 5-to-9 age range, the prevalence of overweight and obesity is 33.5% and 14.3% respectively. The prevalence of overweight among boys more than doubled between 1989 and 2009, from 15% to 34.8%, and that of obesity rose even more markedly in the same period, from 4.1% in 1989 to 16.6% in 2008–2009. The prevalence of overweight and obesity in girls, in turn, rose from 11.9% to 32% and from 2.4% to 11.8% respectively, over the same period [6].

Overweight in childhood is a result of multiple factors [7–9], including absence or short duration of breastfeeding (BF) and exclusive BF (EBF) [10–16], early introduction of complementary foods, and inadequate dietary practices [17–21].

Despite the recommendation of the World Health Organization—EBF in the first six months of life and maintenance of BF for 2 years or more [22]-, in Brazil, the prevalence of EBF in infants under 6 months is low, particularly in children of adolescent mothers (35.8%); the duration of BF is less than one year (median = 11.2 months); and 21% of infants aged 4–6 months eat salty foods, including cereal grains, vegetables, meats and eggs, and 24% eat fruit regularly [23]. Moreover, inadequate dietary practices are common in under-fives [24]. This situation prompted the development of a pro-BF and healthy complementary feeding intervention geared to adolescent mothers and their own mothers (i.e., the infants’ maternal grandmothers), when they cohabited. Grandmothers were included in the intervention due to their potential for negative influence on child feeding practices [25–30]. This intervention, which was tested through a randomized controlled trial, proved to be effective in prolonging the duration of EBF [31] and increasing the prevalence of BF in the first year of life [32], and had a positive impact against early introduction of complementary feeding [33]. In view of the positive results of this intervention and presuming that mode and duration of BF and timing of onset of complementary feeding can influence the future nutritional status of children [10–20], the present study sought to assess the medium-term impact of the same intervention on child growth and prevalence of overweight at age 4–7 years.

## Methods

This randomized clinical trial enrolled 323 adolescent mothers, their infants, and, when living in the same household, their own mothers (that is, the maternal grandmothers of the infants) from May 2006 to January 2008.

All participants were recruited from the rooming-in facility of Hospital de Clínicas de Porto Alegre (HCPA). HCPA is a public general hospital in Porto Alegre, Brazil, and a Baby-friendly Hospital accredited facility where approximately 3,000 deliveries take place per year. On a daily basis, investigators identified all mothers who met the inclusion criteria: age younger than 20 years, lived within Porto Alegre municipal limits, had given birth to a healthy singleton infant with a birth weight of 2,500 g or greater, and had begun BF. Mothers of multiple infants, those who could not room in with their infants due to maternal or neonatal complications, and those who lived with their mothers-in-law (i.e., the child’s paternal grandmother) were excluded from the study. Once identified, adolescent mothers were randomly allocated in blocks of two into the control or intervention groups, i.e., if one mother was randomly allocated to the intervention group, the next eligible mother was automatically allocated to the control group. To ensure the estimated required number of adolescents living with their mothers, it was predetermined that adolescent mothers cohabiting with their own mothers would compose half of the study sample.

Intervention sessions took place in the maternity ward and at each mother’s household, at 7, 15, 30, 60, and 120 days post-delivery. The first session always took place in the maternity ward, 24 to 72 hours after delivery, and consisted of a pro-BF counseling intervention using the communication skills advocated by WHO [34]. In the no-cohabitation group, adolescent mothers alone received the intervention. In the cohabitation group, both mother and grandmother received initial counseling; the initial session was held separately for mothers and grandmothers, on a one-on-one basis. The sessions were led by members of a team composed of two nurses, a dietitian, and a pediatrician, three of whom were International Board Certified Lactation Consultants (IBCLCs). During the first session, the consultant and the mother or grandmother had an informal conversation about several aspects related to BF, with an emphasis on EBF. Supporting material for sessions included booklets and flipcharts designed specifically for the study intervention. All mothers, regardless of group allocation, received standard care as provided at the maternity ward.

When mothers and grandmothers lived in the same household, joint counseling sessions were held. These sessions were used to reinforce messages originally conveyed during initial counseling and to discuss any challenges related to child feeding. The sessions held at 120 days placed emphasis on the introduction of healthy complementary feeding starting at age 6 months, as advocated by the guidelines provided in the *Guia de Alimentação para Crianças Brasileiras Menores de 2 Anos* [35]. During this session, participants were also given brochures with guidance on healthy and timely introduction of complementary feeding.

Data were collected at several time points. At the maternity ward, after agreeing to take part in the study and providing written informed consent, signed by the guardians/caretakers, adolescent mothers and their own mothers (when they cohabited) were interviewed separately to collect sociodemographic data and information on prenatal care, delivery, and prior experience with BF. Data on child feeding during the first year of life were obtained once monthly during the first 6 months and every two months thereafter until 12 months, by means of telephone interviews with the mother or house visits when telephone contact could not be established. Final assessment took place from September 2012 to July 2013, when children were aged 4 to 7 years, at the HCPA Clinical Research Center (or at home when mothers and children failed to attend the center). At this moment, after providing an updated written informed consent, mothers were interviewed to obtain information on current sociodemographic characteristics and child feeding, and duration of breastfeeding. The children were weighed and measured. For the assessment of the dietary intake, we used a not validated food frequency questionnaire, created especially for the study, containing all the food customarily consumed by the studied population, such as vegetables, cereal grains, leguminous, meats, eggs and dairy products.

The consumption of these foods was evaluated in weekly frequency from none to more than five days a week.

For anthropometric assessment, two weight and height measurements were obtained from each child, using the techniques recommended by the Brazilian Ministry of Health [36]. For classification of children by BMI-for-age and height-for-age, the WHO reference populations and cutoff points were used as a standard [37–41]. Data collection and anthropometric assessment were always performed by investigators blinded to group allocation.

Since the original clinical trial was planned to evaluate another question (rates of EBF and BF in the first year of life), we calculated the effect size that can be detected with the sample available at the follow up assessment (n = 207), considering the new question. Thus, estimating a prevalence of overweight of 30% in children aged 4–7 years not exposed to the intervention group, this sample size is sufficient to detect a difference of 20% or more in overweight prevalence among the exposed and unexposed intervention, adopting α = 5% and β = 20%.

All statistical analyses were carried out in SPSS 21.0 for Windows, using the intention-to-treat principle.

Initially, we compared the characteristics of children who were lost to follow-up to those who completed the trial. We then compared the characteristics of the control and intervention groups. The Student *t* or Mann–Whitney *U* tests were used as appropriate for comparison of means, and the Pearson chi-square or Fisher’s exact tests for comparison of proportions. As a result of losses to follow-up, an imbalance between the control and intervention groups was detected for some variables. To make up for this heterogeneity, we used multivariable Poisson regression model with robust variance. We first tested the unadjusted model, and then constructed a series of cumulative models (by sequential addition of new variables) as a result of comparative analysis between groups. The sequential model included those variables with a p-value <0.20, in addition to the propensity score [42, 43]. The propensity scores were estimated using logistic regression, modeling the probability of an individual being allocated to the intervention group and considering the following predictors: maternal age, educational attainment, skin color, and parity; infant weight and mode of delivery; and parental cohabitation. The significance level was set at 5% (p≤0.05).

The study was approved by the HCPA Research Ethics Committee and by Plataforma Brasil (no. 120249), and registered at ClinicalTrials.gov with accession number NCT00910377. The authors confirm that all ongoing and related trials for this drug/intervention are registered.

## Results

**Fig 1:**
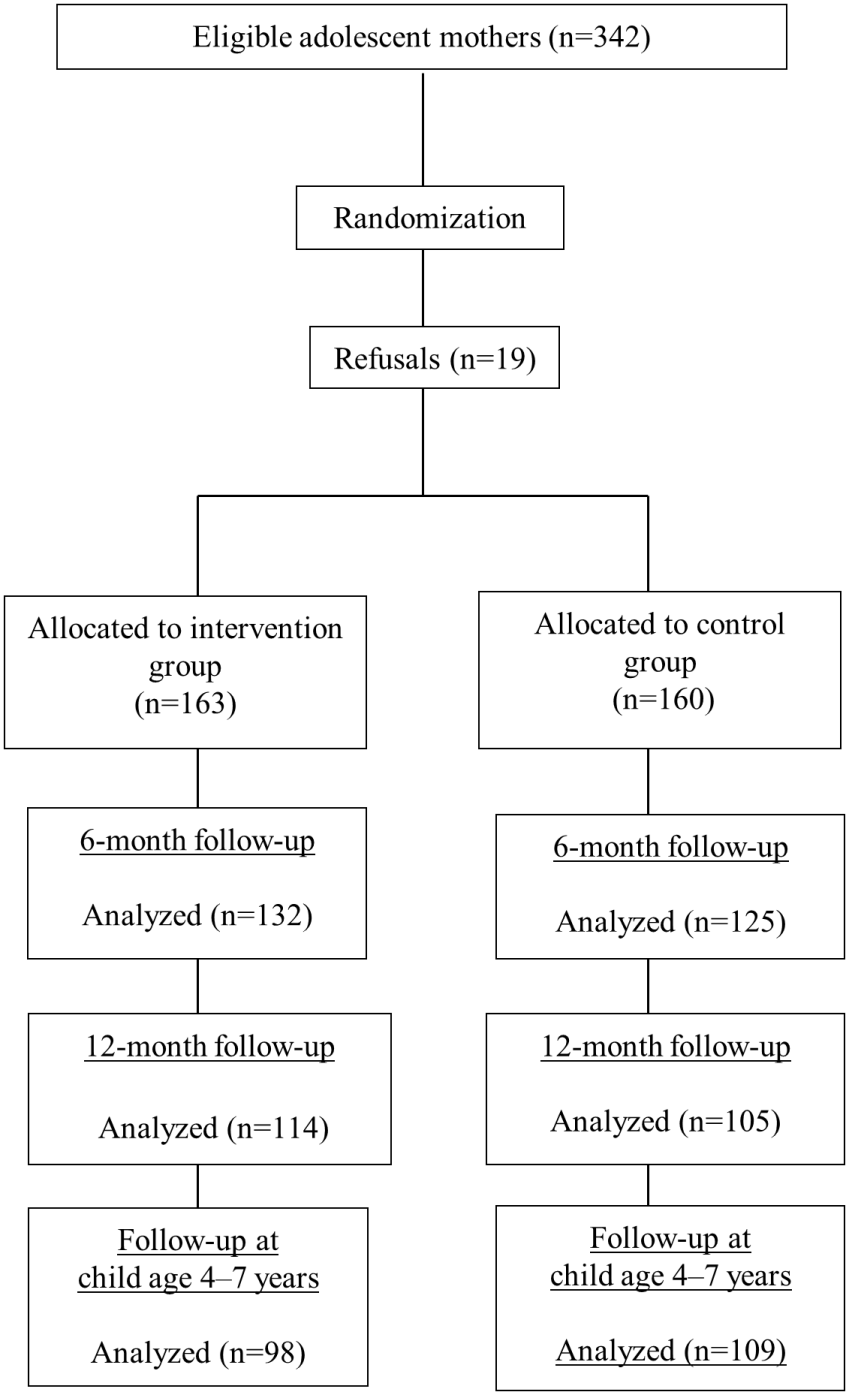
Flow chart of the randomized clinical trial phases from sample selection to the latest assessment, at 4–7 years of age.

Fig 1 shows a flow diagram of the study from recruitment to final assessment, which took place when children were aged 4 to 7 years. Of the 323 mothers/children who started the trial, 207 (64.1%) were located and took part in final assessment.

Table 1 shows that loss to follow-up occurred predominantly in the intervention and no-cohabitation groups, although the differences were not significant. Nevertheless, the intervention and control groups were imbalanced in terms of proportion of cohabitation, current age of the child, and maternal educational attainment (Table 2). The intervention group had a greater proportion of adolescent mothers who lived with their own mothers during the intervention period, a greater proportion of adolescent mothers with ≥8 years of formal schooling and a lower mean child age at final assessment.

**Table 1:** Characteristics of participants who completed the study and of those lost to follow-up.

Variable	Completers (n = 207)	Lost to follow-up (n = 116)	P
Group—n (%)			0.167
Intervention	98 (47.3)	65 (56.0)	
Control	109 (52.7)	51 (44.0)	
Maternal age (years)—mean ± SD	17.5 ± 1.5	17.5 ± 1.5	0.968
Maternal educational attainment, ≥ 8 years—n (%)	110 (53.1)	60 (51.7)	0.898
Infant birth weight (g)—mean ± SD	3.252 ± 424	3.214 ± 385	0.428
Per capita income (MW*)—median (interquartile range)	0.4 (0.3–0.6)	0.4 (0.3–0.7)	0.921
Infant sex, male—n (%)	101 (48.8)	63 (54.3)	0.403
Maternal skin color, white—n (%)	129 (62.3)	74 (63.8)	0.932
Mode of delivery, vaginal—n (%)	154 (74.4)	87 (75.0)	1.000
Primiparity at the time of intervention—n (%)	177 (85.5)	99 (85.3)	1.000
Cohabiting with partner at the time of intervention—n (%)	125 (60.4)	76 (65.5)	0.428
Cohabiting with maternal grandmother at the time of intervention—n (%)	117 (56.5)	52 (44.8)	0.057

SD = standard deviation

*MW: minimum wage (US$195.00 at the time of the study).

**Table 2:** Characteristics of participants who completed the study stratified by group allocation.

Variable	Intervention (n = 98)	Control (n = 109)	P
***At the time of intervention***			
Maternal age (years)—mean ± SD	17.4 ± 1.5	17.5 ± 1.4	0.675
Maternal educational attainment, ≥ 8 years—n (%)	55 (56.1)	55 (50.5)	0.499
Infant birth weight (g)—mean ± SD	3252 ± 421	3252 ± 428	0.995
Per capita income (MW*)—median (interquartile range)	0.5 (0.3–0.6)	0.4 (0.2–0.6)	0.685
Infant sex, male–n (%)	45 (45.9)	56 (51.4)	0.519
Maternal skin color, white—n (%)	62 (63.3)	67 (61.5)	0.902
Mode of delivery, vaginal—n (%)	73 (74.5)	81 (74.3)	1.000
Primiparity—n (%)	88 (89.8)	89 (81.7)	0.143
Cohabiting with partner—n (%)	57 (58.2)	68 (62.4)	0.633
Cohabiting with maternal grandmother—n (%)	64 (65.3)	53 (48.6)	0.023
***At the time of last assessment***			
Maternal age (years)—mean ± SD	23.9 ± 4.0	24.4 ± 1.7	0.305
Child age (years)—mean ± SD	5.82 ± 0.52	6.30 ± 0.36	<0.001
Per capita income (MW)—median (interquartile range)	0.6 (0.4–0.9)	0.6 (0.4–0.8)	0.814
Other children born—n (%)	36 (36.7)	42 (38.9)	0.861
*Bolsa Família* recipient** —n (%)	31 (32.0)	30 (27.8)	0.617
Maternal educational attainment, ≥ 8 years—n (%)	76 (80.9)	72 (67.3)	0.044
Maternal employment outside the home—n (%)	49 (52.1)	63 (58.3)	0.457
Cohabitation with maternal grandmother—n (%)	30 (31.3)	25 (23.4)	0.270
Cohabitation with paternal grandmother—n (%)	3 (3.2)	10 (9.3)	0.138
Cohabitation with partner—n (%)	57 (60.6)	76 (71.0)	0.160

SD = standard deviation

* MW: minimum wage (US$195.00 at the time of the study).

**Bolsa Família is a conditional cash transfer program of the Brazilian federal government whereby benefits are provided to families living in poverty and extreme poverty across the country.

The results of anthropometric assessment are shown in Table 3. The height-for-age and BMI-for-age Z scores were similar between groups. Overall, 38.8% of children in the intervention group and 31.2% of those in the control group had overweight (including obesity), with no significant between-group difference.

**Table 3:** Anthropometric indicators of children at age 4 to 7 years, stratified by group allocation.

Variable	Intervention (n = 98)	Control (n = 109)	P
BMI-for-age–z score	0.87 ± 1.37	0.73 ± 1.33	0.461
Excessive weight (overweight + obesity)—n (%)	38 (38.8)	34 (31.2)	0.318
Overweight*	21 (21.4)	19 (17.4)	
Obesity**	17 (17.3)	15 (13.8)	
Height-for-age—z score	0.12 ± 0.93	-0.01 ± 1.04	0.331
Stunting***	0 (0.0)	3 (2.8)	0.248

BMI = body mass index

*defined as BMI-for-age > +2 z-score and ≤ +3 z-score for children under five; and > +1 z-score and ≤ +2 z-score for older children, according WHO standards

** defined as BMI-for-age > +3 z-score for children under five; and > +2 z-score for older children, according WHO standards

*** defined as length-for-age < -2 z-score, according WHO standards

Data on the child feeding patterns at final assessment are shown in Table 4. The duration of EBF and the age at onset of complementary feeding were significantly greater in the intervention group than in the control group. However, there was no significant difference in the median duration of BF. Furthermore, there were no between-group differences in intake of vegetables, fruit, soft drinks, processed snack foods, fried foods, candy/sweets, cookies, and artificial fruit juices.

**Table 4:** Data on child feeding, stratified by group allocation.

Variable	Intervention (n = 98)	Control (n = 109)	P
*Duration of EBF (months)—median (interquartilerange)	2.9 (1.0–4.7)	1.3 (0.6–3.0)	0.001
*Age at onset of complementary feeding (months)–median (interquartile range)	5 (4–6)	4 (4–6)	0.004
**Duration of BF (months)–median (interquartile range)	12 (4.5–24)	12 (4–24)	0.649
***Food intake vegetables ≥ 5×/week—n (%)	47 (48.0)	46 (42.2)	0.489
fruit ≥ 5×/week—n (%)	55 (56.1)	69 (63.3)	0.363
processed snack foods < 1×/week—n (%)	19 (19.4)	23 (21.1)	0.894
fried foods < 1×/week—n (%)	19 (19.4)	18 (16.5)	0.721
candy/sweets < 1×/week—n (%)	15 (15.3)	15 (13.8)	0.906
cookies < 1×/week—n (%)	26 (26.5)	27 (24.8)	0.896
soft drinks < 1×/week—n (%)	7 (7.1)	8 (7.3)	1.000
****artificial fruit drinks—n (%)	72 (73.5)	93 (85.3)	0.052

EBF = exclusive breastfeeding

BF = breastfeeding

* variables measured through monthly interviews during the first six months of children’s life.

** variable collected through interviews when children were aged 4 to 7 years.

*** variables collected through food frequency questionnaire when children were aged 4 to 7 years.

**** variable collected when children were aged 4 to 7 years and analyzed in order to determine the consumption between groups. The frequency of intake wasn’t measured.

The crude and adjusted effects of intervention on the prevalence of overweight and obesity showed that the study intervention had no impact on overweight and obesity in this sample of children (Table 5).

**Table 5:** Poisson regression model with robust estimation for the effect of intervention on overweight/obesity.

Model	RR (95%CI)	P
1—Intervention group	1.24 (0.86–1.81)	0.254
2—Model 1 + propensity score	1.16 (0.80–1.69)	0.442
3—Model 2 + cohabitation with maternal grandmother at time of intervention	1.16 (0.80–1.70)	0.428
4—Model 3 + maternal educational attainment at final intervention	1.09 (0.74–1.61)	0.675
5—Model 4 + child age	1.11 (0.72–1.70)	0.645
6—Model 5 + cohabitation with paternal grandmother at final intervention	1.11 (0.72–1.70)	0.648
7—Model 6 + cohabitation with partner at final intervention	1.09 (0.70–1.68)	0.703

RR = relative risk

## Discussion

The tested intervention had no effect on nutritional status of children at age 4–7 years, contradicting our initial expectations, which were based on studies showing that increased duration of EBF and BF [13, 16, 44, 45] and later introduction of complementary feeding [9, 17–19, 35, 46–48] are associated with a lower risk of overweight and obesity in childhood. In the present study, children in the intervention group had double the median duration of EBF and a later introduction of complementary feeding than those in the control group; however, this was not enough to influence their nutritional status at age 4–7 years. Although they failed to prove our hypothesis, the results of this study are consistent with those of previous investigations that assessed BF and healthy complementary feeding promotion interventions and had overweight and obesity at preschool age as outcomes of interest [49–52]. Particularly worthy of note is a study conducted in Belarus, by Kramer et al. [49], which was the largest randomized clinical trial to date to test the effect of a BF promotion intervention conducted during the first year of life on a variety of outcomes, including child weight, height, and adiposity at age 12 months and 6.5 years. The BMIs of children in the experiment and control groups at age 6.5 years were similar, as were the proportions of children with overweight (13.4% and 12.2% respectively) and obesity (5.9% and 5.0% respectively). Similar results were found by studies conducted in Bangladesh [52] and London [50], which respectively addressed the impact of a pro-BF intervention in the first 6 months of life and the impact of a dietary practices intervention in the first year of life on nutritional status during preschool age (4–5 years). Furthermore, a previous Brazilian study found no significant differences in the proportions of overweight and obesity between 7- and 8-year-olds whose mothers had received BF counseling during the first year of life and children whose mothers had received no such intervention. The prevalence of overweight and obesity was 31.6% and 15.8% in boys and 29.1% and 12.7% in girls, respectively, in the intervention group, vs. 26.3% and 9.1% in boys and 24.4% and 10.3% in girls, respectively, in the control group [51].

On the basis of some studies that showed that breastfed infants—particularly those breastfed for longer—exhibited healthier dietary habits both in the first year of life and during preschool age as compared with children who had not been breastfed or who had been breastfed for shorter periods [53–57], we expected that children in the present sample would have higher-quality diets after the trial intervention. Conversely, there were no between-group differences in intake of healthy or unhealthy foods.

The absence of any effect of the study intervention on child nutritional status during the preschool years may be attributed to the multitude of factors involved in the genesis of overweight and obesity, such as: maternal obesity in the pre-gestational, gestational, and post-gestational periods; high birth weight; rapid weight gain during the first year of life; maternal smoking; sleep deprivation; TV time; among others [7–9]. Therefore, any intervention seeking to reduce overweight and obesity in children must take these factors into account, and must be sustained, as these factors are dynamic and may change at any point in time. The intervention tested herein took place during the first 4 months of life, and no later “booster sessions” were held. This brief intervention period was sufficient to have an impact on the rates of BF and EBF during the first year of life and on the timing of onset of complementary feeding [31–33], but did not influence later nutritional status.

The merits of this study include its pioneering nature as the first randomized clinical trial to test a pro-BF and healthy complementary feeding intervention in a sample of adolescent mothers and maternal grandmothers, with child nutritional status during preschool age as the outcome. However, some limitations must be noted. Despite exhaustive attempts to locate all families enrolled in the trial, there was a significant rate of loss to follow-up, especially due to participants who moved to unknown locations. High follow-up loss rates are common in population-based prospective studies, particularly those involving young adults living in the peripheral areas of large urban centers in developing countries. To minimize the possibility of selection bias due to attrition, the effect of the intervention on the outcome of interest was adjusted for variables that exhibited between-group differences at the p<0.20 level. It is noteworthy that the losses to follow up during the period between 12 months and the last assessment at 4–7 years were relatively smaller than during the first year, especially if we consider that this is a longer period. In fact, the number of children at the 4–7 years follow-up stage in the control group was higher than the number at 12 months. We believe that this could be possible due to the inclusion of social networks as a search tool of the families for the last assessment, which allowed us to find some families that had been lost before the 12 months arm.

Another possible limitation is the large age range (4 to 7 years) at follow up assessment. It took almost two years to recruit the sample and ten months to locate all families for the follow up assessment. As we did not determine a specific age for the follow up evaluation, we ended up having this wide age range. Yet, we believe that this fact has not affected the results, especially as the child's age was considered in the multivariable analyses.

Regarding the duration of BF, we can not rule out recall bias, as the information was collected retrospectively for mothers breastfeeding for over 12 months (53.2% of the sample). However, this type of bias is more relevant when investigating duration of exclusive breastfeeding [58] as mothers tend to recall the duration of BF with relative accuracy. According to a study conducted in the United States, BF duration was only slightly overestimated at 1 to 3.5 years after the outcome [59]. And finally, we can not disregard the fact that this study did not provide for collection of data that might assist in interpretation of results, such as parental weight and height, infant weight and length during the first year of life, and child physical activity patterns, sleep duration, and TV time.

We conclude that a pro-BF and healthy complementary feeding intervention geared to adolescent mothers and their own mothers (i.e., the maternal grandmothers of the infants) was not effective in preventing child overweight or obesity at preschool age. The multifactorial nature of overweight and obesity in children and the brief intervention period may be implicated in these findings. Nevertheless, even if a longer duration of BF/EBF is not associated with lower prevalence of overweight and obesity later in childhood, other benefits of prolonged BF still stand, such as better metabolic patterns with lower risk of developing heart disease, hypertension, and diabetes [44, 60–63]; superior cognitive development [44]; and benefits for the mother, such as lower risk of breast cancer and type 2 diabetes [64–67]. These benefits mean that promotion of BF should be a priority among health promotion strategies for all nations.

## Supporting Information

S1 CONSORT Checklist CONSORT 2010 Checklist. (DOC)

S1 Protocol Trial study protocol. (DOC)
