# Supplementary material for: The Effect of a Pro-Breastfeeding and Healthy Complementary Feeding Intervention Targeting Adolescent Mothers and Grandmothers on Growth and Prevalence of Overweight of Preschool Children
Source: PLoS One. 2015 Jul 10;10(7):e0131884. doi: 10.1371/journal.pone.0131884 (PMC4498782; doi:10.1371/journal.pone.0131884)
Supplement: S1 Protocol — (DOC) [file pone.0131884.s002.doc]

UNIVERSIDADE FEDERAL DO RIO GRANDE DO SUL - UFRGS

GRADUATE PROGRAM IN HEALTH

CHILD AND ADOLESCENT

Research Project

**IMPACT ON THE MEDIUM TERM OF COUSELING IN BREASTFEEDING AND HEALTHY COMPLEMENTARY FEEDING, CARRIED OUT IN THE FIRST FOUR MONTHS OF CHILD LIFE, IN EATING HABITS AND NUTRITIONAL STATUS: A RANDOMIZED CLINICAL TRIAL INVOLVING ADOLESCENT MOTHERS AND GRANDMOTHERS**

Supervisor: Elsa Regina Justo Giugliani

Co-Supervisor: Luciana Dias de Oliveira

Researchers

Leandro Meirelles Nunes

Lílian Córdova do Espírito Santo

Renata Schwartz

Betina Soldateli

Cristiano Francisco da Silva

Porto Alegre, 2012, June

**1. ABSTRACT**

In view of the low breastfeeding rates (BF) and practical sub-optimal complementary feeding in Brazil; the large number of adolescent mothers in our country; the presumed influence of grandmothers on feeding practices of their grandchildren; the fact that adolescent mothers who live with their mothers (maternal grandmothers of children) are considered to have an increased risk of inadequate feeding practices of their young children; and also the lack of intervention studies with adolescent mothers and grandmothers, it was conducted in the years 2006 to 2008, a randomized clinical trial to evaluate the efficacy of an intervention (six BF counseling sessions and healthy complementary feeding, carried out in the first four months of the child's life, the first in the maternity and the others in households) directed to adolescent mothers and grandmothers on exclusive breastfeeding prevalence (EBF) in the first six months of life and the BF prevalence and adoption of healthy complementary feeding and timely in the first year of life. Due to the favorable results of the intervention on all outcomes studied, it is intended, with this study, knowing the impact of the intervention on dietary habits and nutritional status of children in preschool age and brothers who are born after the intervention. For this, 252 children who were part of the clinical trial will be reassessed at age 4-6 years and younger siblings. Children in the intervention group will be compared with the control group regarding: anthropometric indicators (BMI, height), diet, adequacy of intake of macro and micronutrients as recommended for the age group (Dietary Reference Intakes - DRIs), the duration of BF (preschool for only), the duration of EBF and BF prevalences in the first year of life (only for younger siblings). To quantify the impact of the intervention in the various outcomes will be used Cox regression (hazard-ratio). In the analyzes, will be considered cohabitation with the grandmothers and other relevant variables. Our hypothesis is that the positive impact of the intervention acquired in the first year of life has positively reflected in anthropometric indicators and eating habits of children in preschool and younger siblings.

**2. INTRODUCTION**

The nutritional habits has undergone many changes, affecting mainly the nutritional status of individuals. Breastfeeding (BF) must attended the evolution of the human species in 99.9% of its existence. Despite being biologically determined, this practice, to be under the strong influence of socio-economic and cultural factors, with time has undergone a marked decline. The abandonment of breastfeeding showed, through time, to have harmful consequences for child health, such as high rates of mortality and malnutrition, and also long-term consequences, such as increased predisposition to chronic diseases, including obesity.

Several studies have indicated the BF and EBF as protective factors against overweight / obesity ^1-3^. Three meta-analyzes with observational studies found that the risk of obesity was lower (15-25%) in breastfed infants compared with those who received infant formula^4^.

In the UK, a cohort study evaluated the gain from birth to three years of age of 10,533 children and concluded that the initiation and duration of breastfeeding were significantly associated with weight gain in that period. Infants who did not receive breast milk had higher weight gain than those who were breastfed (z score coefficient: 0.06; 95% CI: 0.02 to 0.09) and those breastfed for less than four months compared with those who were breastfed for longer (z score coefficient: 0.05; 95% CI: 0.01 to 0.09). The authors concluded that initiate and prolong breastfeeding can reduce the excess weight in preschool age ^5^.

In Japan, a cross-sectional study of 448 preschoolers noted that the prevalence of overweight in children who had been exclusively breastfed was 3.8% and in formula-fed infants, 11.8%. However, the EBF until six months was not associated with significant reductions in overweight (OR: 0.70; 95% CI: 0.30 to 1.64) ^6^.

In Brazil, there are few studies related to the association between breastfeeding and overweight / obesity. Balaban et al.,^7^ conducted a cross-sectional study in the city of Recife with 409 children. The authors concluded that children who were breastfed for less than four months had a higher prevalence of overweight compared to those breastfed for longer. Another study conducted in São Paulo, also found a protective effect of breastfeeding against obesity. The authors concluded that children who were never breastfed were twice as likely to develop obesity^8^. And, also in São Paulo, Simon et al.^9^ found through sectional study that EBF for six months and BF for more than 24 months protected against overweight and obesity in a population of 566 children between 2 and 6 years old enrolled in private kindergarten schools.

According to some authors, the protection given to breastfeeding against overweight / obesity is related to the unique composition of human milk, reflecting on the process called metabolic imprinting. This process influence, for example, the number and / or size of adipocytes or inducing metabolic differentiation, occurring in a critical and specific period of development in which early food experiences have lasting effect^10^. Another latest theory is that adiponectin, a protein hormone produced by fat tissue and found in human milk in concentrations above the limit of leptin, would be related to metabolic activities including increased insulin sensitivity, metabolic control and suppression of inflammation, which could explain, at least partially, the protective effect of human milk against overweight / obesity^11^.

In the apparent protection of breastfeeding against overweight / obesity, includes the effect of the introduction of complementary foods in the diet of children. It has been demonstrated association between early consumption of these foods and obesity in childhood and adulthood^12,13^.

Despite evidence of the benefits of EBF, the BF and the healthy complementary feeding, the indicators of BF and complementary feeding in Brazil are sub-optimal, as shown by the II Breastfeeding Prevalence Research in Brazilian capitals and the Federal District held in 2008^14^: median duration of EBF of 52 days with less than 10% of children being exclusively breastfed at six months; BF median duration of 11.2 months, with only 45.5% of children being breastfed for 12 months or more; early onset of complementary feeding, with a quarter of children between three and six months already receiving salty food and / or fruit, 9% consuming biscuits and snacks and almost half receiving other milk between four and six months of life, with 18% receiving this food in the first month of life.

The scientific literature has pointed out several factors related to the early termination of the EBF and BF and early introduction of complementary foods, including the low maternal age and cohabitation with the child's maternal grandmother^15-23^.

Studies in Canada^17^, the US^24^ and Norway^16^ suggest that adolescent mothers are particularly prone to introduce other foods early to the detriment of EBF. In Brazil, a study conducted in the State of São Paulo, involving 33,735 children, found that primiparous women younger than 20 years were 20% higher chance to abandon the EBF before four months^15^. Still in Brazil, Giugliani et al. identified the adolescent motherhood as one of the determinants for the interruption of EBF before six months (HR = 1.48, 95% CI= 1.01 to 2.17)^25^.

There is no doubt that grandmothers, in general, influence the eating habits of young children and can contribute to the success of hers daughters BF^26^. On the other hand, the literature has shown that some grandmothers had a different experience with their own children, and can recommend currently outdated practices such as early use of water, teas, and other milk solids / semi-solids in children under six months.

Some studies conducted in Brazil point to a possible negative influence of grandmothers on feeding their grandchildren in the first months of life. In Natal, Brazil, the prevalence of EBF was significantly lower in children with grandmothers present in the family group^27^. In Porto Alegre, a cohort study of 601 mother-infant pairs found that, regardless of maternal characteristics, only the fact that the maternal or paternal grandmother recommend the introduction of water and / or tea and another type of milk in the diet of baby significantly increased the risk of it not be breastfed exclusively until the end of the first month (OR = 2.22; 95% CI = 1.50 to 3.30 and OR = 1.83, 95% CI = 1,24- 2.71, when the maternal and paternal grandmothers, respectively, recommended water and / or tea and OR = 4.51, 95% CI 2.14 to 9.49 and OR = 1.86; 95% CI = 1, 1 to 3.42 when the maternal and paternal grandmothers, respectively, recommended another type of milk)^19^. Also in Porto Alegre, in another cohort, Giugliani et al. demonstrated that the introduction of water and tea was associated with cohabitation with the maternal grandmother (HR = 1.51; 95% CI 1:10 to 2:10)^25^.

Based on the knowledge described above and considering: low rates of BF and EBF and early onset of complementary foods in Brazil^14^; the large number of adolescent mothers in our country ^28^; the presumed influence of grandmothers on feeding practices of their grandchildren; the fact that adolescent mothers living together with their mothers (maternal grandmothers of children) are considered to have increased risk for inadequate eating habits early in life for their children; and also the lack of intervention studies with adolescent mothers and grandmothers, was conducted in the years 2006 to 2008, a randomized clinical trial to evaluate the efficacy of an intervention (six BF counseling sessions and complementary feeding made in the first four months of the child's life) designed for adolescent mothers and grandmothers in EBF rates in the first six months of life and in breastfeeding rates and adoption of healthy complementary feeding in the first year of life. The main results of this trial are summarized as follows:

- The intervention reduced the unnecessary use of water and / or teas in children breastfed for the first six months by 52% (HR = 0.48, 95% CI 0.31 to 0.76) when it was applied only to adolescent mothers. When the intervention included maternal grandmothers, the protection was 47% (HR = 0.53; CI 95% = 0.35-0.80); and postponed for 44 days the use of these liquids in the group where only the adolescents received the intervention (78 days, 95% CI 50.2 to 105.8 without intervention versus 122 days, 95% CI 107.6 to 136.3 with intervention) and 67 days in the group where the grandmothers were also included in the intervention (63 days, 95% CI 47.2 to 78.7 without intervention versus 130 days, 95% CI 113.4 to 146.6 with intervention^29^.
- The intervention increased the median duration of EBF in 67 days in the group that only adolescent mothers received the intervention (103 days; 95% CI = 82.4 to 123.5 with intervention versus 36 days; 95% CI = 21.5 to 50 5 without intervention) and 46 days in the group where the grandmothers were also included in the intervention (89 days, 95% CI 56.8 to 121.2 with intervention versus 43 days, 95% CI 29.9 to 56.1 without intervention); and reduced by 48% the EBF abandonment in the first half when applied only to adolescent mothers (HR = 0.52; 95% CI = 0.36-0.76) and 36% when included maternal grandmothers (HR = 0,64, 95% CI = 0.46-0.90)^30^.
- The probability of a child be fed with another milk remained lower over the first six months of life in the group receiving the intervention. The median age of introduction of other types of milk was 95 days (95% CI 78.7 to 111.3) in the control group and 153 days (95% CI 114.6 to 191.4) in the intervention group. Therefore, the intervention delayed in nearly two months the introduction of other milks^31^.
- The intervention had a positive impact on the early introduction of complementary foods. At four months, 41% (95% CI 32.8 to 49.2) of the control group were receiving complementary foods. This prevalence decreased to 22.8% (95% CI 15.9 to 29.7) in the intervention group. However, the six-month prevalence rates of children receiving complementary foods were similar in both groups: 88.4% (95% CI 82.9 to 93.9) for the control group and 87.1% (95% CI 81,4 to 92.8) in the intervention group^31^.
- The intervention had significant impact on BF rates only for the group that the grandmother did not live with the mother and the child. The proportion of children breastfed at 12 months in the intervention and control groups that did not cohabitate with her maternal grandmother was 38 and 62% respectively, setting a difference of 24 percentage points; in groups where there was cohabitation with her grandmother, this difference was 8% (41 versus 49%); the risk of weaning in the first 12 months was 49% lower in the group of adolescents who received the intervention and not lived with the grandmothers (HR = 0.51; 95% CI 0.30 to 0.85) and 26% lower the group in which the grandmothers participated in the intervention (HR = 0.74; 95% CI 0.47 to 1.16)^32^.
- The EBF, BF and complementary feeding indicators were similar in both control groups, indicating that cohabitation with maternal grandmothers did not significantly affect these indicators. However, the cohabitation of adolescent and child with her grandmother had a negative influence on the impact of the intervention^29-32^.

Given the concern about the increasing prevalence of overweight / obesity in preschool children and the positive results of multiple counseling sessions in BF and complementary feeding carried out in the first four months of the child's life, for adolescent mothers and grandmothers in the main child feeding indicators in the first year of life, we believe it is relevant to know the impact of this intervention on dietary habits and nutritional status of children in preschool and also of other children who were born after intervention. That was the motivation to carry out this study.

**3. AIM**

**3.1. General aim**

To evaluate the impact in the medium term, of multiple counseling sessions in BF and complementary feeding carried out in the first four months of the child's life, for adolescent mothers and grandmothers when these lived with her daughters on dietary habits and nutritional status children in preschool and younger siblings.

**3.2. Specific aim**

1. Compare anthropometric indicators of preschool and younger brothers of the experimental and control groups.
2. Compare eating habits of preschool and younger brothers of the experimental and control groups.
3. Compare adequacy of intake of macro and micronutrients as recommended for the age group (Dietary Reference Intakes - DRIs) age of preschool and younger brothers of the experimental and control groups.
4. Compare the total duration of breastfeeding of preschool children of the experimental and control groups.
5. Compare the EBF and BF indicators in younger siblings of preschoolers of the experimental and control groups.
6. Compare the performance of the ten steps of healthy eating for children over two years of preschool children in the intervention and control groups.
7. Compare the consumption of unhealthy foods of preschool and younger children in the intervention and control groups.

**4. HYPOTHESIS**

Our hypothesis is that the positive impact of the intervention in the EBF and BF indicators and the adoption of healthy complementary feeding in the first year of life has positively reflected in anthropometric indicators and eating habits of children in preschool and their younger siblings.

To test this hypothesis, children who participated in the first phase of the trial will be evaluated at age 4-7 years and younger siblings, when present, the main outcomes: BMI, height, eating habits and appropriate consumption of macro and micronutrients, as well as total time of BF of the children involved in the first phase of the trial and prevalence and patterns of breastfeeding and eating habits of younger siblings.

**4. METHODS**

This study is a continuation of a randomized clinical trial initiated in 2006 and will be conducted in the city of Porto Alegre, at the Centre for Clinical Research of the Porto Alegre University Hospital and / or families of households whose mothers, children and grandmothers were included in the clinical trial.

The population of this study includes 252 children (and their younger siblings), aged four and seven years, who participated in the clinical trial.

**5.1. Information about the first phase of clinical trial**

For sample size determination, the EpiInfo software was used with the following parameters: α = 5%; β = 20%; ratio between exposed and unexposed = 1: 1; prevalence of EBF in the first month in the group not exposed to the intervention = 56% (ALMEIDA, 1999); difference in the prevalence of EBF in the first month of the unexposed group and the group exposed to the intervention = 25 percentage points. Considering also the same difference between groups (25%) in reducing excess weight in the intervention group of preschool age (KOLETZKO, 2009), it was estimated minimum sample 48 mother-child pairs for each group. Added to this number 10% for losses and 20% for performing multiple regression analysis, totaling approximately 63 participants in each group.

Adolescent mothers, their infants and grandmothers were recruited in the rooming-in facility of the Porto Alegre University Hospital between May 2006 and January 2008, and followed in their homes to children reaching one year of age. Daily, including weekends, mothers were identified that met the following inclusion criteria: age under 20 years, living in the city of Porto Alegre, with healthy babies and birth weight equal to or greater than 2,500 g who had started breastfeeding. Mothers of twins and that, for mother or baby's problems could not stay in rooming and the adolescent mothers living with their mothers in law were not included in the study.

Once identified, mothers were classified into two groups: those who lived and not lived with their mothers (maternal grandmothers of the child). Next, by lottery, they were allocated to the control group (who did not receive the intervention) or the intervention group. In these, when mother and grandmother lived together, both were subjected to intervention; when not cohabiting, only the mother received the intervention. The inclusion of the latter group aimed to evaluate how much of the impact of the intervention could be attributed only to the adolescent mother. All mothers, regardless of the group to which they were allocated, received standard hospital care.

Data collection was performed at different times. In maternity ward, adolescent mothers and grandmothers, after agreeing to participate and signed the written informed consent, were interviewed separately to obtain sociodemographic data and aspects related to prenatal care, childbirth and experience prior to breastfeeding. Separate questionnaires were used for mothers and grandmothers. Information on the children's diets in the first six months of life were obtained monthly by telephone interview with the mother, or home visits in telephone contact impossibility. Interviewers were blind to the group to which they belonged mothers. To check the quality of information, were randomly selected 5% of mothers every month, which were submitted by the researcher coordinator of field, a second interview containing some key questions of follow-up questionnaire.

Intervention sessions occurred in the maternity ward and at home. In the maternity ward, occurred the first counseling session on breastfeeding according to the principles of the World Health Organization^33^, conducted by one of the team members formed by two nurses, a nutritionist and a pediatrician, all with extensive experience in BF, three consultants lactating by the International Board of lactation Consultant Examiners (IBLCE). Interventions in maternity ward were individual and performed separately for the mother and grandmother. In households, the advice was simultaneous.

In counseling sessions, professional, mother and grandmothers dialogued informally about various aspects related to breastfeeding - its importance and recommended duration; Factors that influence the production of milk; breastfeeding technique; pacifier use consequences; crying baby and communication - as well as specific questions for each mother and grandmother were clarified. The mothers were encouraged to breastfeed during the intervention, whenever possible, taking advantage of the opportunity to observe the feeding and guide as to positioning and latch.

As support material, flip charts specially designed for research were used containing information about breastfeeding and prevention and management of common problems. A handbook with similar content to the flipchart was distributed at the end of counseling at the hospital.

In households, mothers and grandmothers, when cohabiting, received sessions of reinforcement counseling when children completed 7, 15, 30, 60 and 120 days. At these sessions, discussed the difficulties faced by mothers and their management, as well as reinforced the messages passed in counseling at the hospital. After 120 days, the intervention also addressed complementary feeding, according to the Guia de Alimentação Para Crianças Brasileiras Menores de Dois Anos ^34^.

**5.2 Methodology of the second phase (current project) study**

Phone contacts will be made with mothers / families to invite the children involved in the first phase of the trial and his younger brothers, when present, to participate in the second phase of the project and request that attend the Clinical Research of the Hospital de Clínicas de Porto Alegre in pre-defined date. Mothers / families who are not found by phone will be visited at home.

Data collection will take place at the Clinical Research Center of the Hospital de Clínicas de Porto Alegre or at home with semi-structured questionnaire to obtain information on the current socio-demographic characteristics and some aspects related to the frequency of children in school preschool or day care; food frequency questionnaire to investigate the consumption of food (the same applied in the first phase of the study) and anthropometric measurements of children.

For the anthropometric measurements of children, will be taken two weight measures with the child barefoot wearing light clothing, positioned in the center of the scale (portable electronic scale with capacity of up to 200kg and sensitivity 50g), upright and distributed weight on both feet; and two height measurements with the barefoot child, standing upright, positioned in the center of the product, with arms extended along the body and the head up, looking at a fixed point at eye level. The heels, shoulders and buttocks should be flat on the stadiometer and internal bones of heels must touch as well as the inside of the knees, and feet must be united making a right angle to the legs. Will be calculated body mass index (BMI = weight [kg] / height^2^) and for the classification of children as the BMI / age and height / age will be used the new curves and WHO parameters^35-37^.

Data collection and anthropometric measurements will be performed by three students graduate, which will remain blinded to the groups they belong to children and their siblings (experimental or control).

The data collected will be stored by double entry, using the Excel program. For statistical analysis, SPSS for Windows is used.

Statistical analyzes will be based on the principle of "intention to treat". Initially, key features will be compared of patients lost to follow-up on those who are evaluated to rule out possible selection bias. The following comparisons of preschool and younger children in the intervention group with the control group will be made:

- Anthropometric indicators (BMI, height) using mean z scores nutritional classification of children.
- Eating habits, using the data to be obtained from the food frequency questionnaire. You may also be compared to the adequacy of diet in the first year of life with current.
- Following the 10 steps of healthy eating for children 2-10 years using the data to be obtained from the food frequency questionnaire.
- Adequacy of macro and micronutrients intake as recommended for the age group (Dietary Reference Intakes - DRIs).
- Total duration of breastfeeding (preschoolers only), using medians.
- Duration of EBF, prevalence of BF and adoption of healthy complementary feeding in the first year of life (for the younger siblings of preschoolers).

To quantify the impact of the intervention in different outcomes, the hazard ratios (HR) and 95% confidence intervals will be calculated by means of Cox regression. In the analyzes, will be considered cohabitation with the grandmothers and other relevant characteristics . Will be adopted p <0.05 as level of statistical significance.

**6. ETHICS CONSIDERATIONS**

This is a study with minimal risk to participants, according to the Research Rules Health (Decree 01/88 of the National Health Congress). Mothers or guardians will be informed about the study and only participate after signing written consent. Will be guaranteed the anonymity of participants and the use of results only for research purposes. The search will start after approval by the Scientific Committee of the Hospital de Clínicas de Porto Alegre. The clinical trial was registered at ClinicalTrials.gov, with the number NCT00910377.

Caretakers will be informed of the results of the assessment and, if necessary, will be advised to seek a health care facility.

**7. SCHEDULE**

| **Data** | **Activity** |
| --- | --- |
| March-June 2012 | Team Training |
| July 2012-April 2013 | Data collection - field work  Double data entry |
| May-June 2013 | Completion of data entry |
| July – October 2013 | Data analysis  Writing, translation and article submission |
| November – december 2013 | Defense of the Master's dissertations |
| January 2014 | Defense of the PHD thesis |

**REFERENCE**

1. Dewey KG. Is breastfeeding prospective against child obesity? J Hum Lact 2003; 19:9-18.
2. Horta BL, Bahl R, Martines JC, Victora CG. Evidence on the long term effects of breastfeeding. Systematic reviews and meta-analyses. World Health Organization. 2007.
3. Novaes JF, Lamounier JA, Franceschini SCC, Priore SE. Efeitos a curto e longo prazo do aleitamento materno na saúde infantil. Nutrire 2009; 34: 139-60.
4. Koletzko B, Kries R, Monasterolo RC, Subías JE, Scaglioni S, Giovannini M, Beyer J, et al. Can infant feeding choices modulate later obesity risk. AM J Clin Nutr 2009; 89: 1002s-8s.
5. Griffiths LJ, Smeeth L, Hawkins SS, Cole TJ, Dezateux C,and the Millennium Cohort Study Child Health Group. Effects of infant feeding practice on weight gain from birth to 3 years. Arch Dis Child published online November 19, 2008. doi: 10.1136/adc.2008.137554.
6. Komatsu H, Yorifuji T, Iwase T, Sasaky A, Takao S, Doi H. Impact of breastfeeding on body weight of preschool children in a rural area of Japan: population-based cross-sectional study. Acta Med Okayama; 2009: 63: 49-55.
7. Balaban G, Silva GAP, Dias MLCM, Dias MCM, Fortaleza GTM, Morotó FMM, et al. O aleitamento materno previne o sobrepeso na infância? Rev Bras Saude Matern Infant 2004; 4: 263-8.
8. Siqueira RS, Monteiro CA. Amamentação na infância e obesidade na idade escolar em famílias de alto nível socioeconômico. Rev Saude Publica 2007; 41: 5-12.
9. Simon VGN, Souza JMP, Souza SB, Aleitamento materno, alimentação complementar, sobre-peso e obesidade em pré-escolares. Rev Saude Publica 2009; 43: 60-9.
10. Balaban G, Silva GAP. Efeito protetor do aleitamento materno contra a obesidade infantil. J Pediatr 2004; 80:7-16.
11. Newburg DS, Woo JG, Morrow AL. Characteristics and potential functions of human milk Adiponectin. The Journal of Pediatrics 2010; 156:s41-s6.
12. Wilson AC, Forsyth JS, Greene AS, Irvine L, Hau C, Howie P. Relation of infant diet to childhood health: seven year follow up of cohort of children in Dundee infant feeding study. BMJ. 1998; 316: 21-5.
13. Wo TC, Chen PH. Health consequences of nutrition in childhood and early infancy. Pediatr Neonatol 2009; 50:135-42.
14. Brasil. Ministério da Saúde. II Pesquisa de prevalência de aleitamento materno nas capitais brasileiras e Distrito Federal. Brasília, DF: Ministério da Saúde; 2009.
15. Venâncio SI, Escuder MML, Kitiko P, Rea MF, Monteiro CA. Frequência e determinantes do aleitamento materno em municípios do Estado de São Paulo. Rev Saude Publica. 2002; 36:313-8.
16. Lande B, Andersen LF, Baerug A, Tygg KU, Lund-Larsen K, Veierod MB, et al. Infant feeding practices and associated factors in the first six months of life: The Norwegian Infant Nutrition Survey. Acta Paediatr, 2003; 92:152-61.
17. Dubois L, Girard M. Social determinants of initiation, duration and exclusivity of breastfeeding at the population level: the results of the Longitudinal Study of Child Development in Quebec (LSCDQ 1998-2002). Can J Public Health. 2003; 94:300-5.
18. Bueno MB, Souza JMP, Souza SB, Paz SMRS, Gimeno SGA, Siqueira AAF. Riscos associados ao processo de desmame entre crianças nascidas em hospital universitário de São Paulo, entre 1998 e 1999: estudo de coorte prospectivo do primeiro ano de vida. Cad Saude Publica. 2003; 19:1453:60.
19. Susin LRO, Giugliani ERJ, Kummer SC. Influência das avós na prática do aleitamento materno. Rev Saude Publica. 2005, 39:141-7.
20. Venâncio SI, Monteiro CA. Individual and contextual determinants of exclusive breast-feeding in São Paulo, Brazil: a multilevel analysis. Public Health Nutr. 2006; 9:40-6.
21. Santo LC, Oliveira LD, Giugliani ER. Factors associated with low incidence of exclusive breastfeeding for de first 6 months. Birth. 2007; 34:212-9.
22. Kerr RB, Berti PR, Chirwa M. Breastfeeding and mixed feeding practices in Malawi: Timing, reasons, decision makers, and child health consequences. Food and Nutrition Bulletin 2007; 28:90-9.
23. Kohlhuber M, Rebhan B, Schwegler U, Koletzko B, Fromme H. Breastfeeding rates and duration in Germany: a Bavarian cohort study. British Journal of Nutrition 2008; 99:1127-32.
24. Carmichael SL, Prince CB, Burr R, Nakamoto F, Vogt RL. Breast-feeding practices among WIC participants in Hawaii. J Am Diet Assoc. 2001; 101:57-62.
25. Giugliani ER, Santo LC, Oliveira LD. Factors associated with low incidence of exclusive breastfeeding for de first 6 months. Birth. 2007; 34:212-9.
26. Grassley JS, Eschiti VS. Two generations learning together: facilitating grandmothers’ support of breastfeeding. IJCE 2005; 22: 23-7.
27. Andrade IGM, Taddei JAAC. Determinantes socioeconômicos, culturais e familiares do desmame precoce numa comunidade de Natal, Brasil. Rev Paul Pediatr 2002; 20:8-18.
28. Brasil. Ministério da Saúde. DATASUS. Rede interagencial de informações para a saúde. Tema do ano: nascimentos no Brasil. RIPSA. Indicadores e dados básicos para a saúde, 2007 (IDB 2007). Disponível em: ˂http://tabnet.datasus.gov.br/cgi/idb2007/tema.pdf˃ (Acessado em 15 ago 2011)
29. Nunes LM, Giugliani ERJ, Espírito Santo LC, Oliveira LD. Reduction of unnecessary intake of water and herbal teas on breastfed infants: a randomized clinical trial with adolescent mothers and grandmothers. J Adolesc Health 2011; 49:258-64.
30. Oliveira LD. Eficácia de uma estratégia de promoção do aleitamento materno e alimentação complementar saudável direcionada a mães adolescentes e avós maternas nas taxas de aleitamento materno exclusivo e na época de introdução dos alimentos complementares e outros leites [Tese]. Porto Alegre: Universidade Federal do Rio Grande do Sul; 2010.
31. Oliveira LD, Giugliani ERJ, Espírito Santo LC, Nunes LM. Impact of a strategy to prevent the introduction of non-breast milk and complementary foods during the first six months of life: a randomized clinical trial with adolescent mothers and grandmothers. Early Hum Develop 2012; 88:357-61.
32. Bica OSC. Impacto do aconselhamento em aleitamento materno na duração dessa prática: um ensaio clínico randomizado envolvendo mães adolescentes e avós [dissertação]. Porto Alegre: Universidade Federal do Rio grande do Sul; 2011.
33. World Health Organization/UNICEF. Infant and young child feeding counseling: an integrated course. Geneva: World Health Organization/UNICEF; 2006.
34. Brasil. Ministério da Saúde. Guia alimentar para crianças menores de 2 anos. Brasília: Ministério da Saúde; 2005. Avaliable at: <http://dtr2001.saude.gov.br/editora/produtos/livros/popup/guia_alimentar_criancas_menores_2anos.htm>
35. De Onis M, Garza C, Victora CG, Bhan MK, Norum K. The WHO Multicentre Growth Reference Study (MGRS): rationale, planning, and implementation. Food Nutr Bull 2004; 25(Suppl):1-89.
36. De Onis M, Onyango A W, Borghi E, Siyam A, Nishida C, Sielmann J. Development of a WHO growth reference for school-aged children and adolescents. Bull WHO 2007;85:660-7.
37. Brasil. Ministério da Saúde. Caderneta de Saúde da Criança. 7ª Ed. Brasília: Ministério da Saúde; 2011.
